# A systematic review of the health-financing mechanisms in the Association of Southeast Asian Nations countries and the People’s Republic of China: Lessons for the move towards universal health coverage

**DOI:** 10.1371/journal.pone.0217278

**Published:** 2019-06-14

**Authors:** Chaw-Yin Myint, Milena Pavlova, Khin-Ni-Ni Thein, Wim Groot

**Affiliations:** 1 Department of Health Services Research, CAPHRI, Maastricht University Medical Center, Faculty of Health, Medicine and Life Sciences, Maastricht University, Maastricht, The Netherlands; 2 Water, Research and Training Center (WRTC), Yangon, Myanmar; 3 Top Institute Evidence-Based Education Research (TIER), Maastricht University, Maastricht, The Netherlands; Tulane University, UNITED STATES

## Abstract

We systematically review the health-financing mechanisms, revenue rising, pooling, purchasing, and benefits, in the Association of Southeast Asian Nations (ASEAN) and the People’s Republic of China, and their impact on universal health coverage (UHC) goals in terms of universal financial protection, utilization/equity and quality. Two kinds of sources are reviewed: 1) academic articles, and 2) countries’ health system reports. We synthesize the findings from ASEAN countries and China reporting on studies that are in the scope of our objective, and studies that focus on the system (macro level) rather than treatment/technology specific studies (micro level).The results of our review suggest that the main sources of revenues are direct/indirect taxes and out of pocket payments in all ASEAN countries and China except for Brunei where natural resource revenues are the main source of revenue collection. Brunei, Indonesia, Philippines, Malaysia, and Viet Nam have a single pool for revenue collection constituting a national health insurance. Cambodia, China, Lao, Singapore, and Thailand have implemented multiple pooling systems while Myanmar has no formal arrangement. Capitation, Fee-for-Service, DRGs, Fee schedules, Salary, and Global budget are the methods of purchasing in the studied countries. Each country has its own definition of the basic benefit package which includes the services that are perceived as essential for the population health. Although many studies provide evidence of an increase in financial protection after reforming the health-financing mechanisms in the studied countries, inequity in financial protection continue to exist. Overall, the utilization of health care among the poor has increased as a consequence of the implementation of government subsidized health insurance schemes which target the poor in most of the studied countries. Inappropriate policies and provider payment mechanisms impact on the quality of health care provision. We conclude that the most important factors to attain UHC are to prioritize and include vulnerable groups into the health insurance scheme. Government subsidization for this kind of groups is found to be an effective method to achieve this goal. The higher the percentage of government expenditure on health, the greater the financial protection is. At the same time, there is a need to weigh the financial stability of the health-financing system. A unified health insurance system providing the same benefit package for all, is the most efficient way to attain equitable access to health care. Capacity building for both administrative and health service providers is crucial for sustainable and good quality health care.

## Introduction

At the fifty-eighth World Health Assembly, in 2005, the member states of the World Health Organization committed themselves to attain universal health coverage (UHC) for their citizens [1]. According to the WHO,”*UHC means that all people receive the health services they need without suffering financial hardship when paying for them*. *The full spectrum of essential*, *quality health services should be covered including health promotion*, *prevention and treatment*, *rehabilitation and palliative care*” [2]. The WHO member states agreed on the development of their health-financing systems by strengthening the role of prepayment for health care while diminishing direct payments, which were seen as one of the barriers to access to health care [3]. The governments’ commitment to achieve UHC was also demonstrated during several high level meetings and resolutions, and finally included as part of goal number 3 in the 2030 Agenda for Sustainable Development. All these agreements highlighted the importance of health in the countries’ development and its high priority in the 21st century [4–7]. Thus, the need for UHC is well recognized. It is expected that UHC can help to reduce out-of-pocket expenses and, at the same time, to provide essential health services to the population, including those for the poor.

Several issues and gaps need to be addressed in moving towards UHC in lower and middle income countries (LMICs). With regard to the legal aspect, UHC simply implies that every resident has access to a basic set of health services. In terms of population coverage and public health, however, it is important to also determine whether UHC succeeds to ensure financial protection, equity and quality of health care across the various population groups, whether the health system financing is sustainable in the long term, and whether the resources for essential health care are used efficiently. It is overall accepted that UHC should not only prevent unaffordable out-of-pocket payments (OOPPs) and major income losses due to the use of health care, but should also eliminate delays in seeking necessary health care for financial reasons [8, 9].

The countries belonging to the Association of Southeast Asian Nations (ASEAN) have started to reform their health financing to attain UHC. These countries face several hurdles to achieve UHC, including a lack of financial and human resources to provide health services. Simultaneously, the ASEAN countries face an increase in health care costs due to non-communicable diseases, persisting infectious diseases, and the reemergence of potentially pandemic infectious diseases [10]. However, each country has chosen a different path of reform depending on the available resources, e.g. external donor support, payroll tax, general revenues. As a result, ASEAN countries have implemented a great variety of health-financing mechanisms in moving towards UHC. The different health-financing mechanisms show a varying level of sustainability, forms of governance and outcomes [11, 12].

The aim of this paper is to systematically review (1) the health-financing mechanisms in ASEAN countries and (2) the impact of these mechanisms on the goals of UHC. The review includes the ASEAN countries: Brunei, Cambodia, Indonesia, Lao PDR, Malaysia, Myanmar, Philippines, Singapore, Thailand, and Viet Nam. We also include China, which is a country close to the ASEAN region, with a similar sociodemographic setting as most ASEAN countries, which has successfully progressed towards UHC in 2011. Our review framework is based on the essential elements of health-financing mechanisms proposed by McIntyre and Kutzin (2016), namely: revenue raising, pooling and purchasing. In addition, we review the level of achievement of UHC goals, utilization (equity in the use of health services), quality of care, and universal financial protection [13].

By systematically reviewing the empirical evidence from the ASEAN countries and China, this paper provides lessons that countries aiming at UHC can learn from experiences in the region. The methods of literature search and analysis are presented in the method section, which is followed by a presentation of the results and their discussion.

## Methods

We reviewed two sources of information: (1) academic journal articles, and (2) countries’ health system reports and website based on the method of systematic literature review [14]. The following chain of keywords used were: *(health insurance system OR social security OR universal health coverage OR insurance agency) AND (payment mechanism OR reimbursement OR accountability OR access to insurance OR solidarity OR efficiency OR health care costs OR quality of care OR progressivity of revenue collection OR regressivity of revenue collection OR cross subsidization OR utilization OR equity) AND (ASEAN OR Brunei OR Brunei Darussalam OR Cambodia OR Indonesia OR Lao PDR OR Lao OR Malaysia OR Myanmar OR Philippines OR Singapore OR Thailand OR Viet Nam OR China)*.

The literature search using the above chain of keywords was conducted in PubMed and Science Direct. The date of the literature search was fixed between 1 Jan 2010 and 11 April 2017. Consequently, our review describes the performance and the characteristics of the health systems in ASEAN countries and China during the period 2006–2017. The papers identified in the systematic search for literature, were checked for their relevance with regard to the review objective defined above. We included all types of studies, quantitative and qualitative studies, as well as mixed methods and reviews. Only English-language papers that reported on empirical studies, were included in the list of relevant publications. Further, the list of papers obtained was limited by a set of criteria. We excluded papers from countries other than ASEAN countries and China, as well as papers reporting on studies that were beyond the scope of our objective, and studies that focused on treatment/technology-specific areas (micro level) other than the system (macro level).

To understand the heterogeneity of the health financing mechanisms, additional information was obtained from the countries’ health system review reports published in the Health System in Transition series. The reports were obtained from the Asia Pacific Observatory on Health Systems and Policies by the World Health Organization Regional Office for the Western Pacific (WPRO). However, no such report was found for Brunei, Singapore and Indonesia. Therefore, we included information obtained from the Singaporean Ministry of Health’s website, the country’s health profile of Brunei, and the countries’ health insurance system report/review of Singapore and Indonesia. The list of references used in the review and type of information provided (health financing mechanism or impact on UHC goals or both), are described in S1 and S2 Files respectively.

The publications (papers and reports) were analyzed by applying the method of directed qualitative content analysis [15]. This analysis is also known as thematic analysis. It requires a selection of key themes in advance and subsequently, extracting and analyzing content related to these themes.

Specifically, our analysis started with the concept of health-financing mechanism by McIntyre and Kutzin (2016) as a guide for selecting themes. The framework outlines the features of a health financing mechanism (revenue raising, pooling, purchasing, benefits) and their influence on the goals and intermediate objectives of UHC. Goals refer to financial protection, quality of care and utilization (equity in the use of health services) while intermediate objectives refer to equity in resource distribution, efficiency as well as transparency and accountability (13). We focus here on the health financing mechanisms and on the UHC goals. To identify the features of the health financing mechanism in a country and to measure the impact of these features on the goals, we identified key indicators (see Table 1). The indicators summarized in Table 1 were the themes used in the analysis.

**Table 1:** The indicators used for applying the framework of McIntyre and Kutzin (2016).

Elements in the frame work	Indicators
Revenue raising	• Direct taxes and Indirect taxes • Non-tax revenues: natural resource revenue • Financing from foreign sources through government • Out-of-pocket
Pooling	• Single pool • Multiple pool
Purchasing	• Type of provider under universal health coverage • Accreditation requirement for providers • Provider payment method ∘ Capitation ∘ Fee-for-Service ∘ DRGs ∘ Fee schedules ∘ Salary ∘ Global budget
Benefits	• Coverage breadth • Coverage scope
Financial protection	• OOPPs • Catastrophic expenditure
Utilization (equity in use of health services)	• Utilization rate among vulnerable group
Quality	• Receiving standard health care • Perceived quality of health care by beneficiaries such as long waiting time.

When screening the full text of the publications reviewed, information related to each theme was extracted from each publication reviewed into an extraction matrix (MS Excel file). Information per theme was synthesized and presented narratively. The narrative description was illustrated by tables.

The quality of all academic journal articles reviewed was assessed by using a quality grading protocol containing assessment items related to key aspects of the research design. Such quality assessment was not possible for the “the Health System in Transition series” and website reviewed because there was no specific explanation of the methods. The assessment tool used for quantitative studies included an assessment of the description of the study design, representativeness of the target population, appropriateness of the research design in relation to the aim of the study, percentage of participation/response, data collection instruments, control for relevant confounders (design/analysis), participants awareness of the research question, appropriateness of the statistical methods, consistency of the intervention, and unintended interventions that might have influenced the results of quantitative studies. The assessment tool used for qualitative studies included an assessment of aim, methodology, appropriateness of the research design in relation to the aim of the study, recruitment, data collection instruments, relationship between researcher and participants, ethical issue, data analysis, clear statement of finding, and value for qualitative studies. For mixed method study, the quantitative part was checked by using the assessment tool for quantitative studies and the qualitative part was checked by using the assessment tools for qualitative studies. The assessment tool used for systematic review study assessed the presence of a focused question, eligibility criteria, literature search description, dual review for determining which studies to include and exclude, quality appraisal for internal validity, list and describe included studies, publication bias, and heterogeneity for systematic review study. The detailed scoring protocol has been described in S3 File. For each item, 1–3 points were given and then an overall average score was calculated as global rating score.

## Results

**Fig 1:**
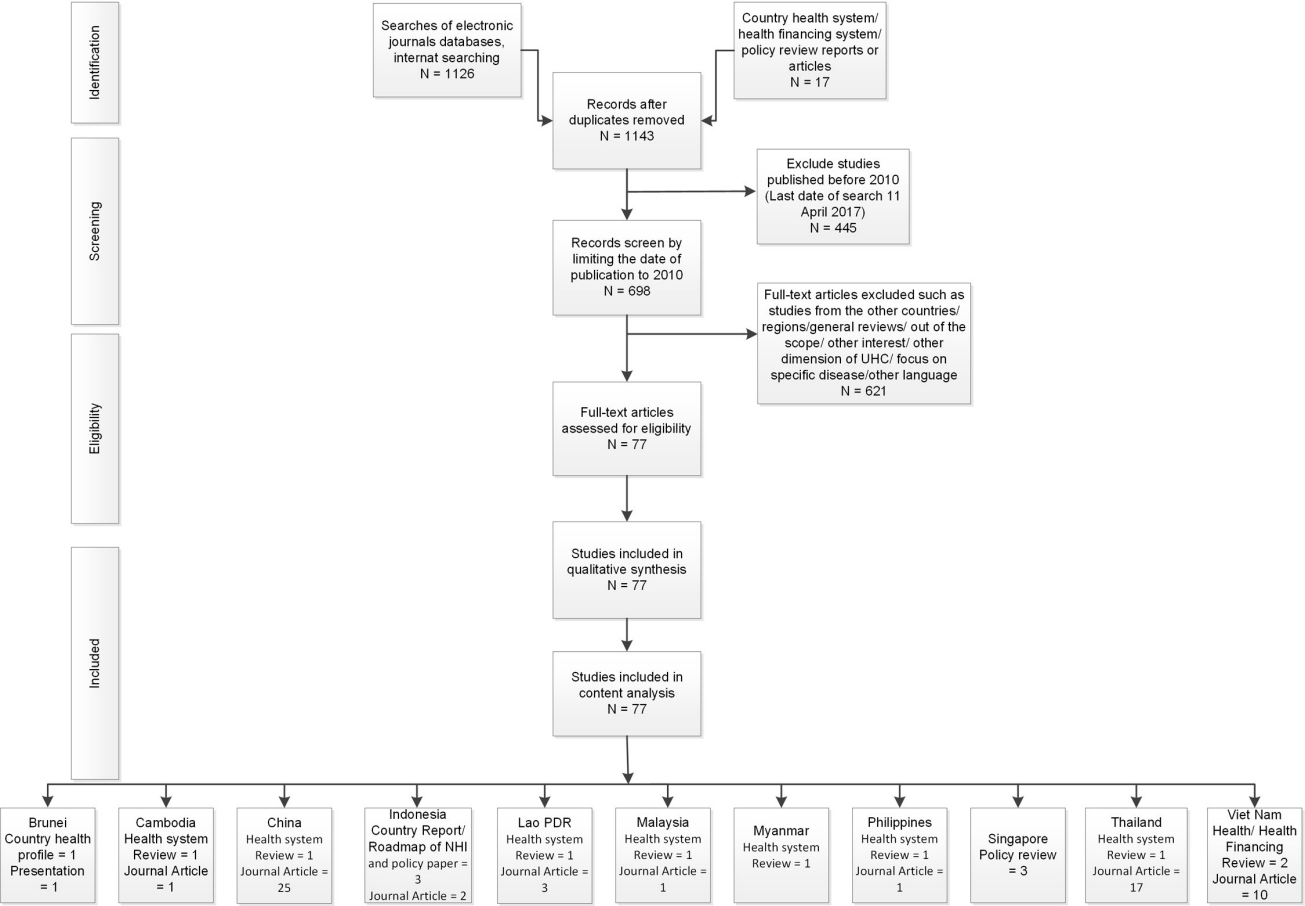
Selection process of the studies.

Fig 1 describes the details of the selection process according to PRISMA guideline. PRISMA 2009 checklist and PRISMA 2009 flow diagram are provided in S4 and S5 Files. The literature search in PubMed and Science Direct resulted in 1126 articles. After excluding articles published before 2010, 681 articles were retained. Then, we excluded 101 articles from countries other than ASEAN countries and China, as well as 467 articles, which were out of the scope of our objective, and 52 studies, which focused on treatment/technology-specific areas (micro level), as well as one study that was not available in English. As a result, 60 articles were included in the systematic review. In addition, 17 country reports were added.

In total, 31 quantitative, 22 qualitative, 6 mixed method and 1 systematic review studies were found. 10 quantitative studies, 10 qualitative studies, 4 mixed-method studies, and one systematic review studies found to be of medium quality and 1 quantitative study found to be of poor quality according to our checklist. The rest were found to be high quality studies.

### Financing mechanisms in the studied countries

The components of health-financing mechanisms such as revenue raising, pooling, purchasing and benefits/ coverage in the ASEAN countries and China are subsequently described in this section.

#### Revenue raising

As the revenue raising mechanism significantly influences the country’s financial protection capacity, our review summarized the ways of revenue raising. The main sources of revenues are direct/indirect taxes and out of pocket payments in all countries except for Brunei where natural resource revenues are the main source of revenue collection [16–22]. The direct taxes include income taxes, pay roll taxes (social health insurance contributions), corporate income or profits taxes, and indirect taxes, i.e. value-added tax (VAT), business tax, and import and export taxes. In Viet Nam. indirect taxes is the major source of revenue [23]. The revenue collection in low ncome countries like Cambodia, Lao PDR and Myanmar include financing by foreign sources and donors through the government [24–26]. Table 2 describes the ways of revenue raising found in the studied countries.

**Table 2:** The ways of revenue raising by country.

Category		Country	Reference index in S1 File.
Revenue raising	Direct taxes and Indirect taxes	Cambodia, China, Indonesia, Lao PDR, Malaysia, Myanmar, Philippines, Singapore, Thailand, Viet Nam	1, 2, 4, 6, 7, 77, 8, 11, 12, 14
	Non-tax revenues: natural resource revenue	Brunei	75,
	Financing from foreign sources through government	Cambodia, Lao PDR, Myanmar	1, 6, 77.
	Out-of-pocket	Cambodia, China, Indonesia, Lao PDR, Malaysia, Myanmar, Philippines, Singapore, Thailand, Viet Nam	1,2, 4, 6, 7, 77, 8, 11, 12, 14

#### Pooling

As indicated in the Table 3, Brunei, Indonesia, Philippines, Malaysia, and Viet Nam have a single pool for revenue collection constituting a national health insurance. Although the revenues go into a single pool, the premium rates vary by employment status in these countries. The exception is Brunei and Malaysia where the single pool is based on direct and indirect general taxes and non-tax revenues other than premium payments [18, 27]. In Indonesia, Philippines and the Viet Nam, the National Health Insurances’ revenues consist of social contribution and government subsidization for the poor people and specific target population such as minority ethnic groups, children under 6 years, civil servants, and other privileged groups [20, 23, 28–30]

**Table 3:** Pooling system of health financing mechanism by country.

Category		Country	Reference index in S1 File.
Pooling	Single pool	Brunei, Indonesia, Philippines, Malaysia, Viet Nam	4, 5, 7, 8, 14, 75
	Multiple pool	Cambodia, China, Lao, Myanmar, Singapore, Thailand	1, 2, 6, 9, 10, 11, 12, 22, 23, 49, 77

Cambodia, China, Lao, Singapore, and Thailand have implemented multiple pooling systems while Myanmar has no formal arrangement. In Cambodia and Lao, the revenues of the health financing system are obtained from employee and employer through social insurance, NGOs through Community-Based Health Insurance, and the government and donors through the Health Equity Funds [24, 25]. In Myanmar, there is no proper arrangement for revenue collection in the health sector except for premium collection in the social security system (SSS) which only covers 1% of the population [26, 31]. The revenues of the Basic Medical Insurance system in China come from premiums collected by the three insurance schemes: one social health insurance, namely Urban Employees’ Basic Medical Insurance (UEBMI); and two community-based health insurances, namely Urban Residents’ Basic Medical Insurance (URBMI) and New Rural Cooperative Medical Scheme (NRCMS). The government of China subsidizes 80% of URBMI and 70% of NRCMS in addition to the individual premium for each scheme. The government of China also funds the National Essential Public Health Services [21, 32, 33].

In Singapore, the “3M” system (Medisave, MediShield Life, Medifund), a special insurance scheme for the elderly, ElderShield, and a government subsidy are the financing mechanisms. Among these multiple tiers, the government subsidies cover up to 80% of the costs of acute hospital care in the first tier of protection to all citizens. In Thailand, the tax-financed mechanism for CSMBS, a mandatory tripartite payroll-tax financing mechanism for the Social Health Insurance Scheme, and general taxes financing mechanism for the Universal Coverage Scheme (UCS) are sources of revenue [19, 34].

#### Purchasing

Table 4 shows the purchasing strategies by country. Service providers for health care in Brunei [27], Cambodia [25], China [21, 33], Lao [24, 35], Malaysia [18], Myanmar [26], and Viet Nam [23, 36] are all public sector providers. However, the health care coverage schemes in Indonesia [20], Singapore [37, 38], Philippines [22] and Thailand [19, 39] rely on a mix of public and private providers. Accreditation is required for the providers in Indonesia [20, 28] and Philippines [22] although it is not required for public providers in Brunei, Cambodia, China, Lao, Malaysia, Myanmar, Singapore, and Viet Nam, and providers in general in Thailand. Details about the type of payment mechanisms can be found in the table.

**Table 4:** Purchasing strategies by country.

Purchasing strategies		Country	Reference index in S1 File.
Type of provider under universal health coverage	Public	Brunei, Cambodia, China, Lao, Malaysia, Myanmar, Viet Nam	1, 2, 6, 7, 14, 22, 65, 75, 77
	Private	-	
	Mixed	Indonesia, Singapore, Philippines, Thailand	4, 8, 9, 10, 12, 54
Accreditation requirement for providers	Yes	Indonesia, Philippines	3, 4, 8,
	No	Brunei, Cambodia, China, Lao, Malaysia, Myanmar, Singapore, Thailand, Viet Nam	1, 2, 6, 7, 9, 12, 14, 75, 77
Provider payment method	Capitation	Cambodia, *China, *Indonesia, *Lao, *Philippine, *Thailand, *Viet Nam	1, 2, 3, 4, 6, 12, 13, 14, 15, 22, 54, 65, 72
	Fee-for-Service	*China, *Malaysia, *Myanmar, *Philippine, *Thailand, *Viet Nam	2, 7, 8, 12, 13, 14, 29, 47, 54, 65, 72, 77
	DRGs	*China, *Indonesia, *Thailand, Viet Nam	2, 3, 4, 12, 14, 49, 54
	Fee schedules	*Indonesia, *Lao	4, 6
	Salary	*Myanmar	65
	Global budget	Brunei, *China, *Malaysia, *Thailand	2, 7, 12, 54,75

*Because of multiple financing scheme and different kinds of health services (outpatient/inpatient or public/private) used, one country may be classified in more than one provider payment system.

#### Benefits/ Coverage

Data on coverage are divided into coverage breadth and coverage scope as describe in Table 5. Coverage breadth means the population groups covered by any form of pre-payment system or social protection while coverage scope refers to the kind of health services provided under a pre-payment system or social protection scheme. With regard to the coverage breadth, more than 95% of the population in China [21] and all citizens in Brunei [16], Malaysia [18], Singapore [37], and Thailand [19] are protected by some form of government subsidies and/or risk-pooling schemes. The coverage breadth in Indonesia (63%) [28], Philippines (76%) [22] and Viet Nam (67%) [30] still have some gaps and have not reached the WHO recommended level of more than 90%. Cambodia (estimated 17–18%) [25], Lao (13.7%) [24], and Myanmar (estimated 1%) [26], have the lowest coverage breadth in the region.

**Table 5:** Benefits/ coverage of currently implemented health-financing mechanism by country.

Category				Country	Reference index in S1 File.
Benefit/ Coverage	Coverage breadth: population protected by government subsidies and/or prepayment and/or risk-pooling schemes	> 90%		Brunei, China, Malaysia, Singapore, Thailand	2, 7, 10, 11, 12, 20, 22, 23, 27, 29, 49, 54, 75
		≤ 90%	90–50%	Indonesia, Philippines, Viet Nam	3, 4, 8, 14, 65
			25–49%	-	
			< 25%	Cambodia, Lao, Myanmar,	1, 6, 77
	Coverage scope: benefit package	Essential health care only		China, Lao, Myanmar, Philippines	2, 6, 8, 20, 22, 23, 29, 77
		Essential health care + high-cost/ tertiary care		Brunei, Indonesia, Malaysia, Singapore, Thailand, Viet Nam	3, 4, 7, 9, 10, 11, 12, 14, 49, 54, 76
		Not defined yet		Cambodia	1

With regard to the coverage scope, it should be pointed out that in Cambodia and Myanmar the Social Health Insurance in both the private and public sector have not defined the benefit package yet [25, 31]. Each country has its own definition of the basic benefit package which includes the services that are perceived as essential for the population health. If a pre-payment system or social protection scheme covers more than the basic package services, the package is described as a generous package. The basic benefit package has been defined in China, Lao, and the Philippines. Because of China’s policy of achieving UHC in a stepwise manner, at the moment only the UEBMI provides a comprehensive service package that covers the costs of outpatient, inpatient, and pharmacy (about 2300 drugs covered). The URBMI and the NRCMS provide limited service packages which mainly cover inpatient services, outpatient services for catastrophic diseases, and limited outpatient services for other diseases [21, 32, 33, 40, 41]. In Lao, the basic package within the Social Health Insurance for the private sector covers outpatient and some inpatient services; and the package within the Social Health Insurance for civil servants covers 50% of total cost for selected services such as transport, major surgery, certain chronic diseases and certain high cost treatments. People enrolled in the CBHI can access health services available at health centers and district hospitals, with referral to provincial and central hospitals if needed. The HEF provides outpatient and inpatient services as well as travel and food subsidies [24]. In the Philippines, the National Health Insurance provides a basic package, which covers expenditures for inpatient services up to a ceiling, as well as expenditures for specific outpatient services such as day surgeries, chemotherapy, radiotherapy, and dialysis [22].

In contrast to these countries, Brunei, Indonesia, Malaysia, Singapore, Thailand, and Viet Nam have a comprehensive benefit package. For example in Brunei and Malaysia, services from prevention and primary health care to tertiary hospital care are completely covered [18, 27, 42]. In Indonesia however, the package is comprehensive but depends on the level of insurance chosen by the individual (of first, second and third-rate care) [20, 28]. In Viet Nam, the SHI provides a comprehensive package based on an inclusive list that covers a broad range of ambulatory and hospital care as well as advanced diagnostic curative health services and therapeutic service [23]. In Thailand and Singapore the coverage is more complex because of the multiple pooling systems. [17, 37, 38], [19, 34, 39]

### The impact of health financing mechanism on UHC

The impact of the health-financing mechanisms in the ASEAN countries and China has been investigated in some of the publications reviewed. The methods used for this include: (1) a comparison of pre and post health-financing mechanisms in terms of financial protection, efficiency, effectiveness, equity, payment and utilization of health services; (2) evaluation of the implementation of the program; review of the implementation of health-financing mechanisms and evaluation. Below, we present the key findings grouped into four categories: financial protection, sustainability, efficiency, equity and quality of health care provision, which are based on the UHC goals as explained at the outset of the paper.

#### Universal financial protection

The aim of UHC is that all people receive the health services they need without suffering financial hardship. Although many studies provide evidence of an increase in financial protection after reforming the health-financing mechanisms in the studied countries, inequity in financial protection continue to exist. The coverage rate in China is almost 100% but there is inequity in financial protection as health care utilization rate among the rich is higher than among the poor. The poor cannot afford the OOPPs required by a policy of high co-payments in China [41, 43–45]. In the Philippines, the poor face uncertainty to pay OOPPs if the hospital charges are higher than the benefit ceiling [46]. Financial protection by insurance in Viet Nam is greater at the district and higher-level state hospitals than at the community health center. We find that charging higher treatment fees and running a private ward for those who can afford to pay, enhance the financial stability of the benefit package. Consequently, there is more financial protection for higher income enrollees who usually seek treatment at the higher-level state hospital. The lower income enrollees usually go to the community health center where OOPPs are higher due to the limited drug coverage, lack of central procurement agency and frequent shortages of drugs [47]. We also find that Thailand achieves a high level of financial protection by providing a comprehensive benefit package resulting in very low incidence of OOPPs and catastrophic health spending [34, 48, 49].

#### Utilization (equity in health care used)

Health service utilization or health outcomes of the target population e.g. poor or vulnerable groups are found to affect equity of health service provision. Sources of revenue collection and patterns of pooling directly impact on equity of health care utilization among insurers. The governments of most ASEAN countries support financial assistance to their population either in the form of subsidization or by establishing free health service provision. A single payer health insurance system with a unified benefit package results in a more equitable health care utilization than fragmented schemes. Overall, the utilization of health care among the poor has increased as a consequence of the implementation of government subsidized health insurance schemes which target the poor e.g., the NRCMS in China [41, 43, 50–53], social health insurance (Askeskin) in Indonesia [54], the national health program in Philippines [46], UCS in Thailand [39, 49, 55–60], and the national health insurance in Viet Nam [47, 61, 62]. The strength of the Malaysian health system is the strong public role in health-financing by protecting the poor and reaching for universal coverage [42]. In Lao, no significant differences in the appropriateness of care for patients at different income levels have been found among the insured patients [35].

In general, value judgements in coverage decisions and the development of well-functioning primary health care facilities can increase equity in health care coverage. For example, equity has been included in the coverage decision process in Thailand, i.e, Renal Replacement Therapy (RTT) has been included in the health benefit package to prevent catastrophic spending and to ensure equity across all schemes despite RRT is not cost-effective and contributed to a long-term fiscal burden [60, 63]. A well-functioning primary health-care system with competent and committed health workers in China and Thailand have been developed to facilitate access to health care especially for the poor [64, 65]. Equity in financial protection has been also improved when fragmented health insurance schemes are consolidated. For example, consolidation of all three fragmented health insurance schemes in China has reduced the inequity in reimbursements between the high-income and low-income population [40].

On the other hand, a fragmented health insurance scheme results either in inequity in access to high quality health care and financial protection or in different health outcomes for different beneficiaries. For example, a huge variation in needs and local fiscal capacity within regions and provinces, and premiums across different health insurance schemes and regions create inequities in insurance coverage in China [32, 41, 44, 52, 66–70]. Similarly, the beneficiaries of different insurance schemes receive care of different quality in Viet Nam due to different reimbursement rates [47]. Another example is Thailand where different health outcomes have been found among the beneficiaries of different insurance schemes particularly in emergency medical care regardless of whether the payment rate is harmonized in all three schemes. Some studies found inequities in inpatient utilization between pro-rich and pro-poor even within the same scheme [55, 58, 71, 72].

Another factor which causes inequity in access to health care is related to high co-payments or premiums or deductibles. For example, in China, the utilization of high quality health services by the rural elderly is low compared to their urban counterpart due to the inability to afford a high co-payment [43, 73–76]. Again in Singapore, the elderly face a lack of insurance coverage (MediShield) for catastrophic health expenses due to age limits, high premiums and deductibles [17].

Regarding factors contributing to inequity in access to health care, automatization of hospitals cannot be overlooked. For example, the financial protection of insurance in Viet Nam is greater at the district and higher-level state hospitals than at the community health center. This is because charging higher treatment fees and running a private ward in these higher hospitals increase financial stability. Consequently, the greater financial protection for the higher income enrollees who usually seek treatment at the higher-level state hospital has been found. The lower income enrollees who usually contact at the community health center where out of pocket payments are higher due to the limited drug coverage, lack of central procurement agency and frequent shortages of drugs [77].

#### Quality

Inappropriate policies and provider payment mechanisms impact on the quality of health care provision. Quality means receiving treatment of needed health care that improves health. For example, GPs in Indonesia have limited professional autonomy in accessing medicines and procedures due to health insurance policies and capitation payment method [78]. Similarly, limitations in transferring patients from rural hospital to tertiary hospitals due to the money follow the patient policies have been implemented in Thailand [58]. The last factor that negatively impacts on quality of health care is a poor referral system from primary care service to tertiary hospital, e.g. in Viet Nam [36].

Some of the studied countries have tried to find ways to improve quality of health care provision and some provide evidence of the impact on quality. Strategic purchasing through an appropriate provider payment mechanism, establishment of a 24 hour call center, and the development of health care accreditation are currently practiced as ways to improve quality of health care. For example, Thailand has arranged for a fee schedule for high cost interventions to prevent the under-provision of services because evidence found that capitation payment causes limited service provision. Thailand has also established a 24-hr call center which receives complaints/problems from beneficiaries and providers to enhance satisfaction of both groups [34]. Moreover, health care accreditation has been developed in Thailand and Indonesia with the expectation that it will contribute to quality of care after adoption of UHC. Although the accreditation process is not mandatory, providers receive an incentive according to their level of accreditation status in Thailand [54, 79].

The perception of insured individuals of the quality of health care provided under the health insurance scheme is crucial for the extension of the health insurance scheme. The perceptions of insured individuals about the quality of health care refer the way of health services provision such as waiting time, consultation time, communications which differ from the definition of health outcome described in above. In Lao and Viet Nam, for example, insured individuals perceived that the quality of health care that they received is poor and includes long waiting times and a limited benefit package [50, 80–82]. This explains the low enrolment rate in Lao and the low utilization rate of health cards to access health services in Viet Nam. The uneven quality of health care can be found not only between different providers (public/private) e.g., Philippines [46], but also within the same facility if the private fee-paying ward is established e.g., hospital automatization in Viet Nam [77].

## Discussion and conclusion

In this paper, we have presented the results of a systematic review of the types of health-financing mechanisms in ASEAN countries and China, and the impact of these mechanisms on achieving UHC goals. Among the 11 countries studied, Brunei and Thailand are the leading countries in moving towards UHC by achieving all three aspects of UHC—coverage breadth, scope and depth or financial protection. As indicated by our review, this is because of the high political will to invest in health and to provide a comprehensive benefit package with negligible or no co-payment by beneficiaries [49]. OOPPs can still be high if pre-paid health insurance systems cannot offer comprehensive benefit services or high co-payments are required either for high cost care (e.g. China and Viet Nam) or private care (e.g. Singapore and Malaysia). GGHE as a percentage of THE in Indonesia and Philippines are nearly equal but Indonesia provides a comprehensive benefit package resulting in a higher financial protection level than the Philippines that has targeted to reach universal coverage breadth.

We found that all three components—revenue raising, pooling and purchasing—of health-financing mechanisms have an effect on equity and quality in health service provision. Specifically, our findings highlight the importance of government investments especially for the poor to extend the coverage scope and financial protection in moving towards UHC. Financial constraints such as low levels of overall and government spending on health care are found to be one of the barriers to achieve UHC in ASEAN countries [10]. For this reason, predominant reliance on public financing in vulnerable population groups is necessary to achieve UHC [83, 84]. On the other hand, in countries where revenues are low, the financial stability of the health system might be endangered by the rise in use and cost of health care like Thailand and Vietnam. Then additional revenues with a high rate of government subsidies may positively impact on the sustainability of the health system.

Another important finding of our review is that a fragmented health-financing systems causes inequity in access to health care, health outcomes, and financial protection among beneficiaries of different health insurance schemes. This result is in line with previous studies on LMICs and BRICs showing that apart from limiting cross-subsidies, the fragmentation of pools has contributed to different benefit packages leading to inequities in access to care, inefficiencies and different level of financial protection across population groups [84, 85]. A consolidation of the fragmented health insurance schemes can improve efficiency and equity in the health system, as shown by one study in selected counties in China [40]. This finding supports previous findings on administrative cost savings through a consolidated system compared to a more fragmented system [86]. However, another study suggested that single-payer models may be the best option for equitable access but it offers limited choice of insurer [87].

Our review also found that the implementation of government provided health insurance schemes generally results in increased health care utilization of the poor. However, some studies in our review pointed out that there is inequity in access to health care not only for beneficiaries of different health insurance schemes but also for the beneficiaries of same health insurance scheme if socioeconomic status and geographic area is not the same. This result supports previous evidence pointing to the greater health care benefits for better-off individuals than the poor especially in hospital expenditure under the same health insurance scheme [88, 89]. In our review, we found studies that report on the greater importance of social values in coverage decisions than scientific evidence in cost-effectiveness [63]. Although this might negatively impact on efficiency, it can increase equity in health care coverage e.g. in Thailand. Moreover, a high cost-sharing policy causes inequity in access to health care among beneficiaries of the same health insurance schemes especially for the poor. This result is in agreement with those obtained by Meng (2011) who found that different levels of cost sharing have an effect on health services utilization. Generally, higher cost sharing leads to lower health care utilization [90].

Our results suggest that capitation payment can improve the efficiency of health insurance systems in ASEAN countries. Specifically, capitation or salary plus performance bonus payments can improve efficiency and CBI performance in developing countries, while fee-for-service may threaten the financial sustainability of CBI schemes [91]. At the same time, our review found that capitation payment can be a limiting factor to provide good quality care. The possible explanation for this is that the provider does not prescribe expensive medicines or diagnostic tests within the limited capitation payment. This result is in accordance with the findings of Gosden et al. (2001), who indicate that fee-for-service resulted in more patient visits, greater continuity of care, and higher compliance with a recommended number of visits. We also found that Thailand has arranged a fee schedule for high cost interventions to prevent the under-provision of services due to capitation payment.

Promoting the integration of accreditation programs in the health insurance system is the best way to improve quality of health care provision as well as contributing to a more standardized method to compare quality of health care provision among providers. Some studies showed that human resource management plays a role in the waiting time at outpatient clinics [92, 93].

The study has some limitations. No country health system or health-financing system report was found for Brunei or Singapore or Indonesia; and no literatures on the impact was found for Brunei or Myanmar or Singapore and no literatures on accountability or transparency of health financing system was found for all 11 studied countries. Only few studies have looked at quality of health care and these studies did not mention how quality is measured which makes it difficult to compare the results.

Our findings show a diversity of implementation strategies of health-financing systems in ASEAN countries and China. Also their impacts on financial protection, utilization (equity in accessing health care) and quality differ. This may provide lessons for the countries on the way to UHC. According to our findings, the most important factor to reach UHC are secure government investments targeting the most vulnerable population groups in addition to revenue collection from the better-off population. At the same time, it should be acknowledged that a reliance on government funds alone can also threaten the sustainability of health financing because any weakening of government commitment can reduce the financial protection for vulnerable people.”. Secondly, a unified health insurance system providing the same benefit package for all is the best way to attain equitable access in health care and more efficient health care provision. Thirdly, capacity building for both administrative and health service providers is crucial for good quality health care. Fourthly, clear guidelines or regulations are needed for the implementation and the coordination between payers, providers and insurers. Finally, a comprehensive benefit package should be offered for the insurance to be attractive and maximize the financial protection.

The authors have no financial, personal or other relationships with other people or organizations that could inappropriately influence to this study.

## Supporting information

S1 File Reference list. (DOCX)

S2 File Reference indexB. (DOCX)

S3 File Quality checklist. (DOCX)

S4 File Prisma checklist. (DOC)

S5 File Prisma diagram. (DOC)
