# Supplementary material for: A systematic review of the health-financing mechanisms in the Association of Southeast Asian Nations countries and the People’s Republic of China: Lessons for the move towards universal health coverage
Source: PLoS One. 2019 Jun 14;14(6):e0217278. doi: 10.1371/journal.pone.0217278 (PMC6568396; doi:10.1371/journal.pone.0217278)
Supplement: S2 File — (DOCX) [file pone.0217278.s002.docx]

S2

Reference Index and type of information provided by each reference

Type of information retrieved for first part of the study “describing health financing mechanisms” or “impact of health financing mechanisms on UHC” or both:

| Ref. no. | Reference | Type of information provided |
| --- | --- | --- |
| 1. . | Peter Leslie Annear, John Grundy, Por Ir, Bart Jacobs, Chean Men, Matthias Nachtnebel, Sophal Oum, Ann Robins and and Chhun Eang Ros (2015). The Kingdom of Cambodia Health System Review. Health System in Transition, Asia Pacific Observatory on Public Health Systems and Policies. | health financing mechanisms |
|  | MENG Qingyue, YANG Hongwei, CHEN Wen, SUN Qiang and and LIU Xiaoyun (2015). People's Republic of China Health System Review. Health System in Transition, Asia Pacific Observatory on Public Health Systems and Policies. | health financing mechanisms |
|  | Mundiharno, Hasbullah Thabrany, Chazali Situmorang, Haris E. Santosa, Fachmi Idris, Ridwan Monoarfa, Timoer Soetanto, Adang Setiana, Moeryono Aladin, Supriyantoro, Djoko Sungkono, Tianggur Sinaga, Bambang Purwoko, Hariyadi B Sukamdani, M. Hutabarat, Sambas Mulyana, Pungky Sumadi, Vivi Yulaswati, Usman Sumantri, Widyastuti, Komarudin, Sensi Wondabio, Imam Supriyadi, Zulkarimien, Yan Kusyanto and and Budi Hidayat (2012). Roadmap toward The National Health Insurance of Indonesia (INA MEDICARE) 2012-2019. | health financing mechanisms |
|  | Puti Marzoeki, Ajay Tandon, Xiaolu Bi and and Eko Setyo Pambudi (2014). Universal Health Coverage for Inclusive and Sustainable Development: Country Summary Report for Indonesia, World Bank Group. | health financing mechanisms |
|  | Mitchell Wiener and I. Muliati (2012). Policy Note on SJSN: Implementation of BPJS Ketenagakerjaan, The World Bank, Program and Policy Implications, Issue 1, May 2012. | health financing mechanisms |
|  | Kongsap Akkhavong, Chanthakhath Paphassarang, Chandavone Phoxay, Manithong Vonglokham, Chansaly Phommavong and and Soulivanh Pholsena (2014). Lao People's Democratic Republic Health System Review. Health System in Transition, Asia Pacific Observatory on Public Health Systems and Policies. | health financing mechanisms |
|  | Safurah Jaafar, Kamaliah Mohd Noh, Khairiyah Abdul Muttalib, Nour Hanah Othman, Judith Healy, Kalsom Maskon, Abdul Rahim Abdullah, Jameela Zainuddin, Azman Abu Bakar, Sameerah Shaikh Abd Rahman, Fatanah Ismail, Chew Yoke Yuen, Nooraini Baba and and Zakiah Mohd Said (2013). Malaysia Health System Review. Health System in Transition, Asia Pacific Observatory on Public Health Systems and Policies. | health financing mechanisms |
|  | Alberto G. Romualdez Jr., Jennifer Frances E. dela Rosa, Jonathan David A. Flavier, Stella Luz A. Quimbo, Kenneth Y. Hartigan-Go, Liezel P. Lagrada and and Lilibeth C. David (2011). The Philippines Health System Review. Health System in Transition, Asia Pacific Observatory on Public Health Systems and Policies. | health financing mechanisms |
|  | Singapore Ministry of Health. (2015). "Ministry of Health, Singapore." Retrieved 15 November 2015, from https://www.moh.gov.sg/content/moh_web/home/costs_and_financing/schemes_subsidies.html. | health financing mechanisms |
|  | Yu BAI, Chaoran SHI, Xiaoteng LI and F. LIU Healthcare System in Singapore: Health Insurance. | health financing mechanisms |
|  | Tilak Abeysinghe, Himani and Jeremy Lim (2010). Singapore's healthcare financing: Some challenges. | health financing mechanisms |
|  | Pongpisut Jongudomsuk, Samrit Srithamrongsawat, Walaiporn Patcharanarumol, Supon Limwattananon, Supasit Pannarunothai, Patama Vapatanavong, Prathom, Krisada Sawaengdee and and Pinij Fahamnuaypol (2015). The Kingdom of Thailand Health System Review. Health System in Transition, Asia Pacific Observatory on Public Health Systems and Policies. | health financing mechanisms |
|  | Nguyen Hoang Long, Sarah Bales, Pham Trong Thanh, Tran Khanh Toan, Duong Duc Thien, Tran Thi Mai Oanh, Nguyen Khanh Phuong, Nguyen Thi Kim Chuc, Hoang Thanh Huong, Khuong Anh Tuan, Nguyen Trong Khoa, Ha Anh Duc, Tran Van Tien, Hoang Van Minh, Nguyen Thanh Huong and and Vu Van Chinh (2013). Joint Annual Health Review 2013: Towards Universal Health Coverage, Ministry of Health, Health Partnership Group. | health financing mechanisms |
|  | Tran Van Tien, Hoang Thi Phuong, Inke Mathauer and Nguyen Thi Kim Phuong (2011). A Health Financing Review of Viet Nam with a focus on Social Health Insurance. | health financing mechanisms |
|  | Annear, P. L., M. Bigdeli and B. Jacobs (2011). "A functional model for monitoring equity and effectiveness in purchasing health insurance premiums for the poor: evidence from Cambodia and the Lao PDR." Health Policy 102(2-3): 295-303. | health financing mechanisms |
|  | Sun, J., X. T. Zhang, Z. Zhang and H. V. Hogerzeil (2014). "Achieving universal health coverage-the case of Zhuhai city." J Evid Based Med 7(3): 154-162. | impact of health financing mechanisms on UHC |
|  | Li, C., Y. Hou, M. Sun, J. Lu, Y. Wang, X. Li, F. Chang and M. Hao (2015). "An evaluation of China's new rural cooperative medical system: achievements and inadequacies from policy goals." BMC Public Health 15: 1079. | impact of health financing mechanisms on UHC |
|  | Dai, B., J. Zhou, Y. J. Mei, B. Wu and Z. Mao (2011). "Can the New Cooperative Medical Scheme promote rural elders' access to health-care services?" Geriatr Gerontol Int 11(3): 239-245. | impact of health financing mechanisms on UHC |
|  | Shi, L. and D. Zhang (2013). "China's new rural cooperative medical scheme and underutilization of medical care among adults over 45: evidence from CHARLS pilot data." J Rural Health 29 Suppl 1: s51-61. | impact of health financing mechanisms on UHC |
|  | Meng, Q., H. Fang, X. Liu, B. Yuan and J. Xu (2015). "Consolidating the social health insurance schemes in China: towards an equitable and efficient health system." Lancet 386(10002): 1484-1492. | health financing mechanisms |
|  | Liu, J. Q. (2011). "Dynamics of social health insurance development: examining the determinants of Chinese basic health insurance coverage with panel data." Soc Sci Med 73(4): 550-558. | impact of health financing mechanisms on UHC |
|  | Yip, W. C., W. C. Hsiao, W. Chen, S. Hu, J. Ma and A. Maynard (2012). "Early appraisal of China's huge and complex health-care reforms." Lancet 379(9818): 833-842. | health financing mechanisms |
|  | Sun, J., S. Deng, X. Xiong and S. Tang (2014). "Equity in access to healthcare among the urban elderly in China: does health insurance matter?" Int J Health Plann Manage 29(2): e127-144. | health financing mechanisms |
|  | Xin, H. (2015). "Experiences and Lessons from Urban Health Insurance Reform in China." Popul Health Manag. | impact of health financing mechanisms on UHC |
|  | Fang, K., B. Shia and S. Ma (2012). "Health insurance coverage and impact: a survey in three cities in China." PLoS One 7(6): e39157. | impact of health financing mechanisms on UHC |
|  | Yuan, S., C. Rehnberg, X. Sun, X. Liu and Q. Meng (2014). "Income related inequalities in New Cooperative Medical Scheme: a five-year empirical study of Junan County in China." Int J Equity Health 13: 38. | impact of health financing mechanisms on UHC |
|  | Zhang, Y., W. Tang, X. Zhang, Y. Zhang and L. Zhang (2015). "National Health Insurance Development in China from 2004 to 2011: Coverage versus Benefits." PLoS One 10(5): e0124995. | both |
|  | Chen, M., W. Chen and Y. Zhao (2012). "New evidence on financing equity in China's health care reform--a case study on Gansu province, China." BMC Health Serv Res 12: 466. | both |
|  | Yu, H. (2015). "Universal health insurance coverage for 1.3 billion people: What accounts for China's success?" Health Policy 119(9): 1145-1152. | health financing mechanisms |
|  | Zhang, L., X. Cheng, X. Liu, K. Zhu, S. Tang, L. Bogg, K. Dobberschuetz and R. Tolhurst (2010). "Balancing the funds in the New Cooperative Medical Scheme in rural China: determinants and influencing factors in two provinces." Int J Health Plann Manage 25(2): 96-118. | both |
|  | Zhang, Z., J. Wang, M. Jin, M. Li, L. Zhou, F. Jing and K. Chen (2014). "Can medical insurance coverage reduce disparities of income in elderly patients requiring long-term care? The case of the People's Republic of China." Clin Interv Aging 9: 771-777. | both |
|  | Dai, T., H. P. Hu, X. Na, Y. Z. Li, Y. L. Wan and L. Q. Xie (2016). "Effects of New Rural Cooperative Medical Scheme on Medical Service Utilization and Medical Expense Control of Inpatients: A 3-year Empirical Study of Hainan Province in China." Chin Med J (Engl) 129(11): 1280-1284. | impact of health financing mechanisms on UHC |
|  | Yu, B., Q. Meng, C. Collins, R. Tolhurst, S. Tang, F. Yan, L. Bogg and X. Liu (2010). "How does the New Cooperative Medical Scheme influence health service utilization? A study in two provinces in rural China." BMC Health Serv Res 10: 116. | impact of health financing mechanisms on UHC |
|  | Flato, H. and H. Zhang (2016). "Inequity in level of healthcare utilization before and after universal health coverage reforms in China: evidence from household surveys in Sichuan Province." Int J Equity Health 15: 96. | both |
|  | Zhou, Z., J. Gao, A. Fox, K. Rao, K. Xu, L. Xu and Y. Zhang (2011). "Measuring the equity of inpatient utilization in Chinese rural areas." BMC Health Serv Res 11: 201. | impact of health financing mechanisms on UHC |
|  | Li, C., X. Yu, J. R. Butler, V. Yiengprugsawan and M. Yu (2011). "Moving towards universal health insurance in China: performance, issues and lessons from Thailand." Soc Sci Med 73(3): 359-366. | both |
|  | Ma, J., J. Xu, Z. Zhang and J. Wang (2016). "New cooperative medical scheme decreased financial burden but expanded the gap of income-related inequity: evidence from three provinces in rural China." Int J Equity Health 15: 72. | both |
|  | Liu, X., H. Wong and K. Liu (2016). "Outcome-based health equity across different social health insurance schemes for the elderly in China." BMC Health Serv Res 16: 9. | both |
|  | Li, X. and W. Zhang (2013). "The impacts of health insurance on health care utilization among the older people in China." Soc Sci Med 85: 59-65. | impact of health financing mechanisms on UHC |
|  | Dai, B. (2015). "The old age health security in rural China: where to go?" Int J Equity Health 14: 119. | both |
|  | Syah, N. A., C. Roberts, A. Jones, L. Trevena and K. Kumar (2015). "Perceptions of Indonesian general practitioners in maintaining standards of medical practice at a time of health reform." Fam Pract 32(5): 584-590. | impact of health financing mechanisms on UHC |
|  | Sparrow, R., A. Suryahadi and W. Widyanti (2013). "Social health insurance for the poor: targeting and impact of Indonesia's Askeskin programme." Soc Sci Med 96: 264-271. | both |
|  | Alkenbrack, S., B. Jacobs and M. Lindelow (2013). "Achieving universal health coverage through voluntary insurance: what can we learn from the experience of Lao PDR?" BMC Health Serv Res 13: 521. | both |
|  | Alkenbrack, S., K. Hanson and M. Lindelow (2015). "Evasion of "mandatory" social health insurance for the formal sector: evidence from Lao PDR." BMC Health Serv Res 15: 473. | both |
|  | Syhakhang, L., D. Soukaloun, G. Tomson, M. Petzold, C. Rehnberg and R. Wahlstrom (2011). "Provider performance in treating poor patients--factors influencing prescribing practices in lao PDR: a cross-sectional study." BMC Health Serv Res 11: 3. | both |
|  | Chua, H. T. and J. C. Cheah (2012). "Financing universal coverage in Malaysia: a case study." BMC Public Health 12 Suppl 1: S7. | impact of health financing mechanisms on UHC |
|  | Tobe, M., A. Stickley, R. B. del Rosario, Jr. and K. Shibuya (2013). "Out-of-pocket medical expenses for inpatient care among beneficiaries of the National Health Insurance Program in the Philippines." Health Policy Plan 28(5): 536-548. | health financing mechanisms |
|  | Mate, K. S., A. L. Rooney, A. Supachutikul and G. Gyani (2014). "Accreditation as a path to achieving universal quality health coverage." Global Health 10: 68. | impact of health financing mechanisms on UHC |
|  | Tangcharoensathien, V., S. Limwattananon, W. Patcharanarumol, J. Thammatacharee, P. Jongudomsuk and S. Sirilak (2015). "Achieving universal health coverage goals in Thailand: the vital role of strategic purchasing." Health Policy Plan 30(9): 1152-1161. | health financing mechanisms |
|  | Suriyawongpaisal, P., W. Aekplakorn and R. Tansirisithikul (2015). "Does harmonization of payment mechanisms enhance equitable health outcomes in delivery of emergency medical services in Thailand?" Health Policy Plan 30(10): 1342-1349. | impact of health financing mechanisms on UHC |
|  | Sripen Tantivess, Román Pérez Velasco, Jomkwan Yothasamut, Adun Mohara, Hatai Limprayoonyong and Yot Teerawattananon (2012). "Efficiency or equity: value judgments in coverage decisions in Thailand." Journal of Health Organization and Management 26(3): 331-342. | impact of health financing mechanisms on UHC |
|  | Ruangratanatrai, W., S. Lertmaharit and P. Hanvoravongchai (2015). "Equity in health personnel financing after Universal Coverage: evidence from Thai Ministry of Public Health's hospitals from 2008-2012." Hum Resour Health 13: 59. | impact of health financing mechanisms on UHC |
|  | Yiengprugsawan, V., G. Carmichael, L. Y. Lim, S. Seubsman and A. Sleigh (2011b). "Explanation of inequality in utilization of ambulatory care before and after universal health insurance in Thailand." Health Policy Plan 26(2): 105-114. | impact of health financing mechanisms on UHC |
|  | Damrongplasit, K. and G. Melnick (2015). "Funding, coverage, and access under Thailand's universal health insurance program: an update after ten years." Appl Health Econ Health Policy 13(2): 157-166. | health financing mechanisms |
|  | Tangcharoensathien, V., S. Limwattananon, R. Suphanchaimat, W. Patcharanarumol, K. Sawaengdee and W. Putthasri (2013). "Health workforce contributions to health system development: a platform for universal health coverage." Bull World Health Organ 91(11): 874-880. | impact of health financing mechanisms on UHC |
|  | Panpiemras, J., T. Puttitanun, K. Samphantharak and K. Thampanishvong (2011). "Impact of Universal Health Care Coverage on patient demand for health care services in Thailand." Health Policy 103(2-3): 228-235. | impact of health financing mechanisms on UHC |
|  | Somkotra, T. (2011). "Measurement and explanation of horizontal (in)equity in health care utilization among Thais after universal coverage policy implementation." Asia Pac J Public Health 23(6): 980-995. | impact of health financing mechanisms on UHC |
|  | Youngkong, S., R. Baltussen, S. Tantivess, A. Mohara and Y. Teerawattananon (2012). "Multicriteria decision analysis for including health interventions in the universal health coverage benefit package in Thailand." Value Health 15(6): 961-970. | impact of health financing mechanisms on UHC |
|  | Tangcharoensathien, V., S. Pitayarangsarit, W. Patcharanarumol, P. Prakongsai, H. Sumalee, J. Tosanguan and A. Mills (2013). "Promoting universal financial protection: how the Thai universal coverage scheme was designed to ensure equity." Health Res Policy Syst 11: 25. | impact of health financing mechanisms on UHC |
|  | Intaranongpai, S., D. Hughes and S. Leethongdee (2012). "The provincial health office as performance manager: change in the local healthcare system after Thailand's universal coverage reforms." Int J Health Plann Manage 27(4): 308-326. | impact of health financing mechanisms on UHC |
|  | Teerawattananon, Y., N. Tritasavit, N. Suchonwanich and P. Kingkaew (2014). "The use of economic evaluation for guiding the pharmaceutical reimbursement list in Thailand." Z Evid Fortbild Qual Gesundhwes 108(7): 397-404. | health financing mechanisms |
|  | Thoresen, S. H. and A. Fielding (2011). "Universal health care in Thailand: concerns among the health care workforce." Health Policy 99(1): 17-22. | impact of health financing mechanisms on UHC |
|  | Mee-Udon, F. (2014). "Universal Health Coverage Scheme impact on well-being in rural Thailand." Int J Health Care Qual Assur 27(6): 456-467. | impact of health financing mechanisms on UHC |
|  | Yiengprugsawan, V., G. A. Carmichael, L. L. Lim, S. A. Seubsman and A. C. Sleigh (2010a). "Has universal health insurance reduced socioeconomic inequalities in urban and rural health service use in Thailand?" Health Place 16(5): 1030-1037. | impact of health financing mechanisms on UHC |
|  | Phuong, N. K., T. T. Oanh, H. T. Phuong, T. V. Tien and C. Cashin (2015). "Assessment of systems for paying health care providers in Vietnam: implications for equity, efficiency and expanding effective health coverage." Glob Public Health 10 Supppl 1: S80-94. | health financing mechanisms |
|  | Liu, X., S. Tang, B. Yu, N. K. Phuong, F. Yan, D. D. Thien and R. Tolhurst (2012). "Can rural health insurance improve equity in health care utilization? A comparison between China and Vietnam." Int J Equity Health 11: 10. | impact of health financing mechanisms on UHC |
|  | Nguyen, H. and J. Knowles (2010). "Demand for voluntary health insurance in developing countries: the case of Vietnam's school-age children and adolescent student health insurance program." Soc Sci Med 71(12): 2074-2082. | impact of health financing mechanisms on UHC |
|  | Sepehri, A. (2014 a). "Does autonomization of public hospitals and exposure to market pressure complement or debilitate social health insurance systems? Evidence from a low-income country." Int J Health Serv 44(1): 73-92. | impact of health financing mechanisms on UHC |
|  | Sepehri, A. (2014 b). "How much do I save if I use my health insurance card when seeking outpatient care? Evidence from a low-income country." Health Policy Plan 29(2): 246-256. | impact of health financing mechanisms on UHC |
|  | Nguyen, K. T., O. T. Khuat, S. Ma, D. C. Pham, G. T. Khuat and J. P. Ruger (2012). "Impact of health insurance on health care treatment and cost in Vietnam: a health capability approach to financial protection." Am J Public Health 102(8): 1450-1461. | impact of health financing mechanisms on UHC |
|  | Do, N., J. Oh and J. S. Lee (2014). "Moving toward universal coverage of health insurance in Vietnam: barriers, facilitating factors, and lessons from Korea." J Korean Med Sci 29(7): 919-925. | impact of health financing mechanisms on UHC |
|  | Ha, B. T., S. Frizen, M. Thi le, D. T. Duong and D. M. Duc (2014). "Policy processes underpinning universal health insurance in Vietnam." Glob Health Action 7: 24928. | health financing mechanisms |
|  | Nguyen, H. and W. Wang (2013). "The effects of free government health insurance among small children--evidence from the free care for children under six policy in Vietnam." Int J Health Plann Manage 28(1): 3-15. | impact of health financing mechanisms on UHC |
|  | Guindon, G. E. (2014). "The impact of health insurance on health services utilization and health outcomes in Vietnam." Health Econ Policy Law 9(4): 359-382. | impact of health financing mechanisms on UHC |
|  | World Health Organization (2011). Brunei Darussalum's Country Health Information Profile. | health financing mechanisms |
|  | Joshua McKinney (2013). Brunei Universal Healthcare. | health financing mechanisms |
|  | Than Tun Sein, Phone Myint, Nilar Tin, Htay Win, San San Aye and and T. Sein (2014). The Republic of the Union of Myanmar Health System Review. Health System in Transition, Asia Pacific Observatory on Public Health Systems and Policies. | health financing mechanisms |
