# Supplementary material for: A systematic review of the health-financing mechanisms in the Association of Southeast Asian Nations countries and the People’s Republic of China: Lessons for the move towards universal health coverage
Source: PLoS One. 2019 Jun 14;14(6):e0217278. doi: 10.1371/journal.pone.0217278 (PMC6568396; doi:10.1371/journal.pone.0217278)
Supplement: S3 File — (DOCX) [file pone.0217278.s003.docx]

**S3**

**Quality Check List**

Quality Check List for Quantitative studies

| Ref. number in Appendix A | Description of study design (clearly stated each step=3; not enough information=2; not described=1) | Representative of the target population (well representative sampling method and sample size=3; Limited sample size=2; not enough sample size=1) | Research Design appropriate to the aims of the study (results answer research questions=3;= results are not enough to answer the research question 2; results are not relevant to the research question =1) | Percentage of participation  (≥ 80% = 3; 80-70% = 2; ≤ 70% =1) | Data collection tools (relevant and validated=3; poor validity/reliability score=2; no information=1) | Control for relevant confounders(design/analysis) (taking control measure for cofounders=3; not properly control=2; no control=1) | Participants aware of the research question (participants were properly informed=3; active communication with participant=2; no information=1) | Statistical methods appropriate or not (appropriate method=3; applicable but not the best method=2; not appropriate=1) | Consistency of the intervention (consistency persist throughout the study period=3; some degree of consistency=2; no consistency=1) | Received an unintended intervention that may influence the results (prevent unintended intervention=3; not properly prevent=2; no information=1) | Global rating score  (3 = strong;  2 = medium; 1 = weak) |
| --- | --- | --- | --- | --- | --- | --- | --- | --- | --- | --- | --- |
| 15. | 3 | 2 | 2 | 3 | 3 | 1 | NA | 3 | 3 | 2 | 3 |
| 19. | 3 | 2 | 3 | 3 | 3 | 1 | 2 | 3 | 3 | 2 | 3 |
| 21. | 3 | 3 | 2 | 3 | 2 | 2 | NA | 2 | 3 | 2 | 2 |
| 23. | 3 | 3 | 3 | 3 | 3 | 1 | NA | 3 | 3 | 2 | 3 |
| 25. | 3 | 3 | 3 | 3 | 3 | 1 | 3 | 3 | 3 | 2 | 3 |
| 26. | 3 | 3 | 3 | 3 | 3 | 3 | 1 | 3 | 3 | 2 | 3 |
| 27. | 3 | 3 | 3 | 3 | 3 | 1 | NA | 3 | 3 | 2 | 3 |
| 28. | 3 | 3 | 2 | 3 | 3 | 1 | 3 | 3 | 3 | 2 | 3 |
| 31. | 3 | 2 | 3 | 2 | 3 | 3 | 2 | 3 | 3 | 1 | 3 |
| 32. | 1 | 1 | 1 | 3 | 1 | 1 | 1 | 2 | 3 | 1 | 1 |
| 34. | 2 | 2 | 2 | NC | 2 | 1 | 1 | 3 | 3 | 2 | 2 |
| 35. | 3 | 3 | 3 | NC | 1 | 1 | 1 | 3 | 3 | 2 | 2 |
| 37. | 3 | 2 | 2 | 3 | 3 | 1 | 2 | 3 | 3 | 2 | 2 |
| 38. | 2 | 3 | 3 | 3 | 3 | 2 | 1 | 3 | 3 | 2 | 3 |
| 39. | 3 | 2 | 3 | NC | 3 | 2 | 1 | 3 | 3 | 2 | 2 |
| 42. | 3 | 3 | 2 | 2 | 3 | 1 | 1 | 3 | 3 | 2 | 2 |
| 44. | 3 | 2 | 2 | 2 | 3 | 1 | 3 | 3 | 3 | 2 | 2 |
| 45. | 3 | 2 | 3 | 3 | 3 | 1 | 3 | 3 | 3 | 2 | 3 |
| 47. | 2 | 2 | 2 | 3 | 2 | 1 | 3 | 3 | 3 | 3 | 2 |
| 50. | 3 | 3 | 3 | 3 | 3 | 1 | NA | 3 | 3 | 3 | 3 |
| 52. | 3 | 3 | 3 | 3 | 3 | 1 | NA | 3 | 3 | 3 | 3 |
| 53. | 3 | 2 | 2 | 2 | 3 | 1 | 3 | 3 | 3 | 2 | 2 |
| 54. | 3 | 3 | 2 | 3 | 2 | 1 | 3 | 3 | 3 | 3 | 3 |
| 56. | 3 | 3 | 3 | 3 | 3 | 1 | NA | 3 | 3 | 2 | 3 |
| 57. | 3 | 3 | 3 | 3 | 3 | 3 | 3 | 3 | 3 | 2 | 3 |
| 64. | 1 | 3 | 3 | NC | 1 | 1 | 1 | 3 | 3 | 2 | 2 |
| 67. | 3 | 2 | 3 | 3 | 2 | 1 | 3 | 3 | 3 | 2 | 3 |
| 69. | 3 | 3 | 2 | 3 | 3 | 2 | 3 | 3 | 3 | 2 | 3 |
| 70. | 3 | 2 | 3 | 3 | 2 | 1 | 3 | 3 | 3 | 3 | 3 |
| 73. | 3 | 3 | 3 | 3 | 3 | 1 | 2 | 3 | 3 | 3 | 3 |
| 74. | 3 | 3 | 2 | 3 | 2 | 2 | 2 | 3 | 3 | 3 | 3 |

Quantitative studies – 31; High quality – 20; Medium quality -10; Poor quality -1

Quality Check List for Qualitative studies

| Ref. number in Appendix A | Aim (well informed objective of the study=3 not enough information=2; not described=1) | Methodology (match a particular research project=3; poorly match=2; not match=1) | Research Design appropriate to the aims of the study (results answer research questions=3; results are not enough to answer the research question=2; results are not relevant to the research question =1) | Recruitment (representative to the aim of study=3; limited recruitment=2; not representative=1) | Data collection tools(relevant and validated=3; poor validity/reliability score=2; no information=1) | Relationship between researcher and participants (participants were properly informed about research=3; active communication with participant=2; no information=1) | Ethical issue (clearly explained ethical consideration=3; not clear=2; no information=1) | Data Analysis (properly searched cofounding factors=3; limited searched cofounding factors=2; no search=1) | Clear statement of findings (well stated findings e.g. meaning, views =3; not clearly stated findings=2; no enough findings=1) | How valuable (high impact to society e.g feasible for implementation=3; moderate impact e.g. some limitation to implement=2; low impact e.g. difficult to implement=1) | Global rating score  (3=strong;  2=medium; 1=weak) |
| --- | --- | --- | --- | --- | --- | --- | --- | --- | --- | --- | --- |
| 1-14, 75-78 | NA (Health System Reports) and relevant web site/presentations | | | | | | | | | | |
| 16. | 3 | 2 | 2 | NA | 2 | NA | NA | 2 | 3 | 3 | 2 |
| 20. | 3 | 3 | 3 | NA | 2 | NA | NA | 2 | 3 | 3 | 3 |
| 22. | 3 | 2 | 3 | NA | 2 | NA | NA | 3 | 2 | 2 | 2 |
| 24. | 3 | 1 | 2 | NA | 1 | NA | NA | 1 | 2 | 2 | 2 |
| 29. | 3 | 2 | 2 | NA | 2 | NA | NA | 3 | 3 | 2 | 2 |
| 36. | 2 | 2 | 2 | NA | 2 | NA | NA | 1 | 2 | 2 | 2 |
| 40. | 3 | 2 | 2 | NA | 3 | NA | NA | 2 | 3 | 3 | 3 |
| 41. | 3 | 3 | 3 | 2 | 3 | 3 | 3 | 3 | 3 | 2 | 3 |
| 46. | 3 | 2 | 2 | NA | 3 | NA | NA | 3 | 3 | 3 | 3 |
| 48. | 3 | 2 | 3 | NA | 2 | NA | NA | 3 | 3 | 3 | 3 |
| 49. | 3 | 3 | 3 | NA | 2 | NA | NA | 3 | 3 | 3 | 3 |
| 51. | 3 | 2 | 3 | NA | 3 | NA | 1 | 2 | 3 | 2 | 2 |
| 55. | 3 | 2 | 2 | NA | 2 | NA | NA | 2 | 3 | 3 | 2 |
| 58. | 3 | 3 | 3 | NA | 3 | NA | NA | 3 | 3 | 3 | 3 |
| 59. | 3 | 3 | 3 | 2 | 3 | 2 | 3 | 3 | 3 | 3 | 3 |
| 60. | 3 | 2 | 3 | 2 | 3 | 3 | 1 | 3 | 3 | 3 | 3 |
| 61. | 3 | 2 | 2 | NA | 2 | NA | NA | 2 | 3 | 3 | 2 |
| 62. | 3 | 3 | 3 | 2 | 3 | 2 | 1 | 2 | 3 | 3 | 3 |
| 65. | 3 | 2 | 3 | 3 | 2 | 3 | 2 | 3 | 3 | 3 | 3 |
| 68. | 3 | 2 | 2 | NA | 2 | NA | NA | 2 | 3 | 3 | 2 |
| 71. | 3 | 1 | 1 | NA | 1 | NA | NA | 1 | 3 | 3 | 2 |
| 72. | 3 | 3 | 3 | 2 | 3 | 3 | 1 | 3 | 3 | 3 | 3 |

Qualitative studies – 22; High quality – 12; Medium quality -10; Poor quality -0

Quality Check List for Mixed studies

| Ref. number in Appendix A | Description of study design | Representative of the target population | Research Design appropriate to the aims of the study | Percentage of participation | Data collection tools | Control for relevant confounders (design/analysis) | Participants aware of the research question | Statistical methods appropriate or not | Consistency of the intervention | Received an unintended intervention that may influence the results | Global rating score  (3=strong;  2=medium; 1=weak) |
| --- | --- | --- | --- | --- | --- | --- | --- | --- | --- | --- | --- |
|  | Aim | Methodology |  | Recruitment |  | Relationship between researcher and participants | Ethical issue | Data Analysis | Clear statement of findings | How valuable |  |
| 17. | 3 | 3 | 2 | 3 | 3 | 1 | 2 | 3 | 3 | 2 | 2 |
|  | 3 | 2 | 2 | 3 | 3 | 2 | 3 | 2 | 2 | 2 |  |
| 30. | 3 | 2 | 2 | 3 | 3 | 1 | 2 | 2 | 3 | 2 | 2 |
|  | 2 | 2 | 3 | 3 | 3 | 3 | N/A | 3 | 3 | 2 |  |
| 33. | 3 | 2 | 3 | 2 | 3 | 2 | 2 | 3 | 3 | 2 | 3 |
|  | 2 | 2 | 3 | 3 | 2 | 3 | N/A | 3 | 2 | 3 |  |
| 43. | 3 | 2 | 2 | 3 | 3 | 1 | 1 | 3 | 3 | 2 | 2 |
|  | 3 | 2 | 2 | 2 | 3 | 2 | 3 | 3 | 2 | 3 |  |
| 63. | 2 | 2 | 3 | 3 | 2 | 1 | 3 | 3 | 3 | 2 | 2 |
|  | 3 | 3 | 3 | 2 | 2 | 2 | 1 | 3 | 3 | 3 |  |
| 66. | 3 | 2 | 3 | 3 | 3 | 1 | 2 | 3 | 3 | 3 | 3 |
|  | 3 | 3 | 3 | 2 | 3 | 3 | 1 | 3 | 3 | 3 |  |

*scales are same as check-list tables for quantitative and qualitative studies

Mixed studies – 6; High quality – 2; Medium quality -4; Poor quality -0

Quality Check List for Systematic reviews

| Ref. number in Appendix A | Focused question  (research question, objective and inclusion criteria established before the review=3; not clearly set prior research objective=2; no information=1) | Eligibility criteria (clearly defined eligibility criteria according to research objective=3; not relevant to research objective=2; no information=1) | Literature search (comprehensive e.g. at least 2 sources + 1 supplementary=3; 2 sources or 1 sources+ 1 supplementary=2; only one source/ no information=1) | Dual review for determining which studies to include and exclude ( 2 people do study selection, 2 people do data extraction, do consensus or different person checks the process=3; only one person do study selection and data extraction=2; no information=1) | Quality appraisal for internal validity (quality scoring tool or checklist=3; only summary score/range of all studies=2; no information=1) | List and describe included studies (provide list of included studies and excluded studies=3; provide only list of included studies=2; no information=1) | Publication bias | Heterogeneity | Global rating score  (3=strong;  2=medium; 1=weak) |
| --- | --- | --- | --- | --- | --- | --- | --- | --- | --- |
| 18. | 2 | 3 | 3 | 2 | 1 | 2 | NA | NA | 2 |
