# Supplementary material for: A systematic review of the health-financing mechanisms in the Association of Southeast Asian Nations countries and the People’s Republic of China: Lessons for the move towards universal health coverage
Source: PLoS One. 2019 Jun 14;14(6):e0217278. doi: 10.1371/journal.pone.0217278 (PMC6568396; doi:10.1371/journal.pone.0217278)
Supplement: S5 File — (DOC) [file pone.0217278.s005.doc]

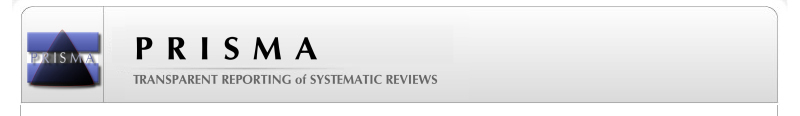
**PRISMA 2009 Flow Diagram**

**Screening**

**Included**

**Eligibility**

**Identification**

Records identified through database searching
(n = 1126)

Additional records identified through other sources
(n = 17)

Records after duplicates removed
(n = 1143)

Records screened
(n = 698)

Records excluded
(n = 445)

Full-text articles assessed for eligibility
(n = 77)

Full-text articles excluded, other countries/ regions/ general review, out of scope, focus on specific disease or other dimension of UHC, other language
(n = 621)

Studies included in qualitative synthesis
(n = 77)

Studies included in content analysis
(n = 77)
